# Multimodal Imaging-Based Phenotyping of a Singaporean Hospital-Based Cohort of High Myopia Patients

**DOI:** 10.3389/fmed.2021.670229

**Published:** 2022-01-04

**Authors:** Kai Yuan Tey, Quan V. Hoang, Isabella Q. Loh, Yee Shan Dan, Qiu Ying Wong, Daryle Jason G. Yu, Vivi R. Yandri, Marcus Ang, Gemmy C. M. Cheung, Shu Yen Lee, Tien Yin Wong, SNEC Retina Group, Anna Cheng, Rachel S. Chong, Chee Wai Wong

**Affiliations:** ^1^Singapore Eye Research Institute, Singapore National Eye Centre, Duke-NUS Medical School, Singapore, Singapore; ^2^Department of Ophthalmology, Columbia University College of Physicians and Surgeons, New York, NY, United States; ^3^Department of Ophthalmology, Yong Loo Lin School of Medicine, National University of Singapore, Singapore, Singapore

**Keywords:** high myopia, pathologic myopia, multimodal imaging, myopic macular degeneration, myopic traction maculopathy

## Abstract

**Purpose:** To assess the effect of axial length (AL) on the prevalence of pathologic myopia (PM) and associated myopic features in a Singaporean hospital-based cohort of patient with high myopia (HM).

**Methods:** In total, 923 HM eyes from 495 individuals were recruited from the Myopic and Pathologic Eyes in Singapore (MyoPES) cohort and underwent ocular biometry, fundus photography, fundus autofluorescence, and swept-source optical coherence tomography (SS-OCT). Images were analyzed for the presence of myopic macular degeneration (MMD), myopic choroidal neovascularization (mCNV), myopic traction maculopathy (MTM), peripapillary atrophy (PPA), myopic tilted disc, posterior staphyloma (PS), dome-shaped macula (DSM), vitremacular adhesions (VMA), and the epiretinal membrane (ERM). Eyes were stratified into quartiles based on ALs to determine cut-off values to perform comparisons between shorter-length and longer-length groups. A χ^2^-test was done to determine the difference in the prevalence of pathologies between groups.

**Results:** Overall, mean AL was 29.2 ± 2.2 mm (range 25.0–36.7 mm). Myopic macular degeneration, PPA, myopic tilted disc, and ERM have AL threshold of ≥27.5 mm, whereas MTM has an AL threshold of ≥29.0 mm. We found that there was a significantly higher prevalence of MMD (88.2 vs. 49.4%; *p* < 0.001), PPA (98.1 vs. 80.1%; *p* < 0.001), myopic tilted disc (72.7 vs. 50.2%; *p* < 0.001), and ERM (81.4 vs. 17.3%; *p* = 0.003) in eyes with AL ≥ 27.5 mm vs. eyes without AL <27.5 mm. Prevalence of MTM (34.7 vs. 32.1%; *p* < 0.001), mCNV (17.4 vs. 12.1%; *p* = 0.03), PS (43.4 vs. 34.7%; *p* = 0.012), DSM (21.3 vs. 13.2%; *p* = 0.002), and VMA (5.9 vs. 2.6%; *p* = 0.014) in eyes with AL ≥ 29.0 mm compared with AL < 29.0 mm.

**Conclusion:** Our study describes the overall prevalence of PM and related pathologies among patients with HM in our hospital-based cohort. Longer eyes even among HM eyes had a significantly higher prevalence of PM-associated pathologies studied. This supports the premise that eyes with longer AL, even among HM eyes may be at greater risk of vision-threatening changes and therefore merit regular follow-up.

## Introduction

Myopia is the leading cause of distance refractive error in the world, affecting 1.89 billion of the global population in 2017 (1), and it is believed that this number will continue to grow due to the myopia boom as a result of a modern lifestyle (2). Mild-to-moderate myopia is a common condition, often due to axial elongation, that can be corrected with appropriate spectacles, contact lenses, and refractive surgery (3).

High myopia, on the other hand, due to excessive ocular elongation (4) can predispose the eye to a risk of permanent vision loss through the development of pathologic myopia (PM), which often manifests clinically as one or a combination of the following conditions: myopic macular degeneration (MMD) (5, 6), myopic choroidal neovascularization (mCNV), or myopic traction maculopathy (MTM) (6–12). While the WHO defines high myopia as spherical equivalent (SE) of five diopters (D) of myopia and above (13), definitions of high myopia vary in the current literature, with most defining high myopia as ≥5.0–8.0 D of myopia (14–16), or axial length (AL) 25–27 mm and above (17–20). High myopia is the second major cause of vision impairment worldwide, having affected approximately 2.8% (170 million) of the global population, with the highest prevalence in East and Southeast Asia, such as China, Japan, the Republic of Korea, and Singapore (2, 13). The prevalence of high myopia continues to grow rapidly, and it is estimated that by 2050, 9.8% (938 million) people globally will be highly myopic (HM) (16). Consequently, the prevalence of PM-related visual impairment is likely to rise in tandem with the prevalence of high myopia (21, 22).

In addition to PM, HM eyes are more likely to develop associated myopic pathologies, such as peripapillary atrophy (PPA) (23), myopic tilted disc (24), posterior staphyloma (PS) (25), dome-shaped macula (DSM) (26), vitreomacular adhesion (VMA) (27), and the epiretinal membrane (ERM) (28). Determining the characteristics of patients with HM at high risk of developing these pathologic changes is critical in guiding the optimal monitoring of patients with HM, so as to administer timely interventions to mitigate or prevent permanent visual loss (29).

In this study, we aimed to assess the prevalence of the aforementioned myopia-related pathologies in a cohort of hospital-based patients with HM using multimodal imaging, including widefield swept-source optical coherence tomography (SS-OCT) and fundus photography/autofluorescence. To further evaluate the impact of AL on PM (13, 30), we stratified patients into two groups based on a cut-off threshold derived from quartile analysis in the hopes of providing credence to an AL-based system to guide the frequency of clinical follow-up for patients with HM.

## Methods

We conducted a cross-sectional analysis of high myopes from the Myopic and Pathologic Eyes in Singapore (MyoPES) cohort. In brief, high myopes defined by an SE refractive error of ≥5.0 D of myopia and/or AL ≥ 25.0 mm in the study eye, and aged 18 years old and above were enrolled from the High Myopia clinic at the Singapore National Eye Centre, Singapore, from January 2017 to December 2018. The study was performed with approval from the SingHealth Institutional Review Board and in accordance with the Declaration of Helsinki. Written informed consent was obtained from all participants. Participants with any existing or previous ocular diseases in either eye that may confound measurements from SS-OCT, and fundus photography were excluded from the study. Such conditions included corneal opacities, uveitis, dense cataracts, vitreous hemorrhage, diabetic retinopathy/diabetic macular edema, central serous chorioretinopathy, previous retinal laser photocoagulation or photodynamic therapy, retinal dystrophies, and macular scarring from any cause other than myopic maculopathy, retinopathy due to any cause other than myopia, previous retinal vein or artery occlusion, and ocular ischemic syndrome.

All enrolled participants underwent the following investigations: (1) ocular biometry, (2) dilated retinal examination, (3) color fundus photography, (4) fundus autofluorescence, and (5) SS-OCT.

### Image Acquisition and Assessment

Axial length was measured using Aladdin HW 3.0, Topcon (Topcon Medical Systems, Oakland, NJ, USA). Triton DRI OCT Plus (Topcon Medical Systems, Oakland, NJ, USA) was used to obtain both fundus photos and SS-OCT images after pupillary dilation. Two fields of each eye were photographed, with one centered at the optic disc and another centered at the fovea. For SS-OCT, images of the fovea, optic disc, vitreous, and sclera were obtained. Scans performed included fovea-centered 3D raster (12 × 9 mm), 3D disc (6 × 6 mm), and fovea-centered radial (diameter 12 mm) scans.

All images and scans obtained were assessed by two retinal specialists (QVH and CWW) for the presence of MMD that includes plus signs (such as mCNV), MTM, myopic tilted disc, PPA, PS, DSM, VMA, and ERM.

**Figure 1:**
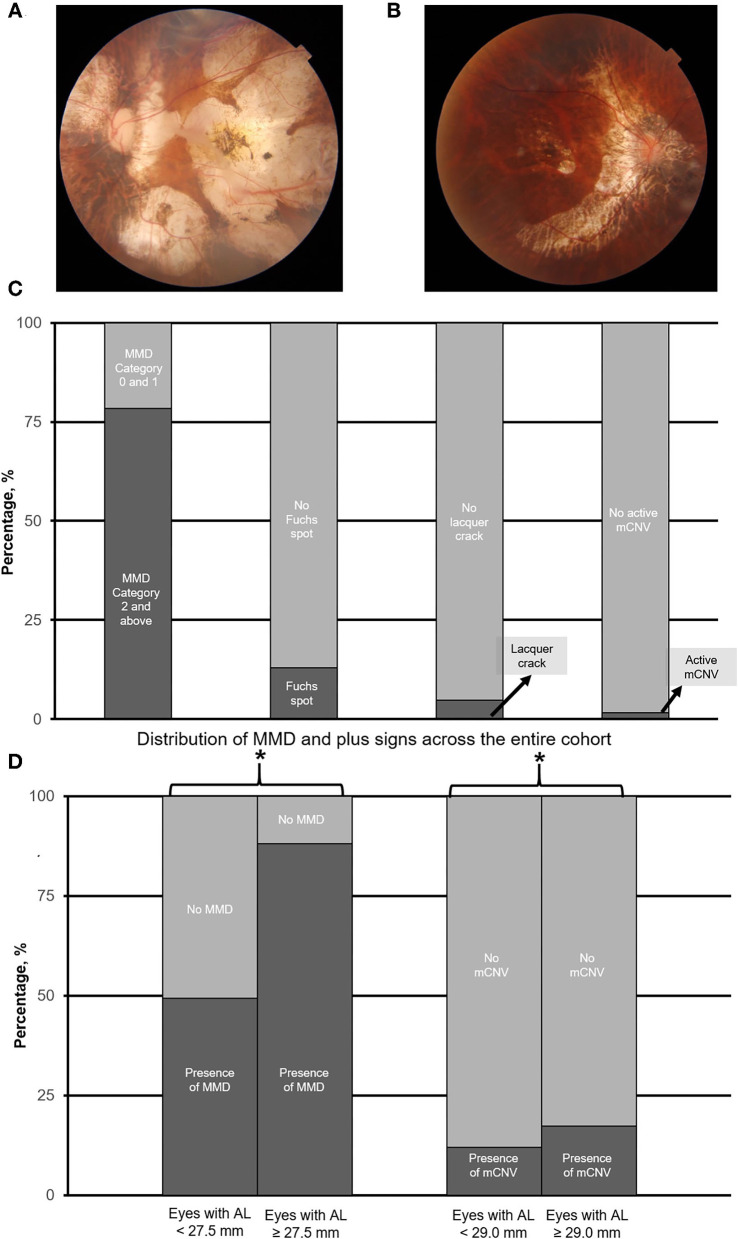
**(A)** and **(B)** are the representative fundus photograph of a myopic macular degeneration (MMD) category 4, i.e., macular atrophy, and active myopic choroidal neovascularization (mCNV), respectively. Panel **(C)** shows the overall prevalence of myopic macular degeneration as assessed by the META-PM classification system. Panel **(D)** illustrates the prevalence of MMD (including plus signs), and mCNV in eyes with axial length (AL) 25.0–27.5 mm vs. with AL ≥ 27.5 mm, and AL 25.0–<29.0 mm vs. AL ≥ 29.0 mm, respectively. Asterisk (*) denotes statistical significance (*p* < 0.05).

**Figure 2:**
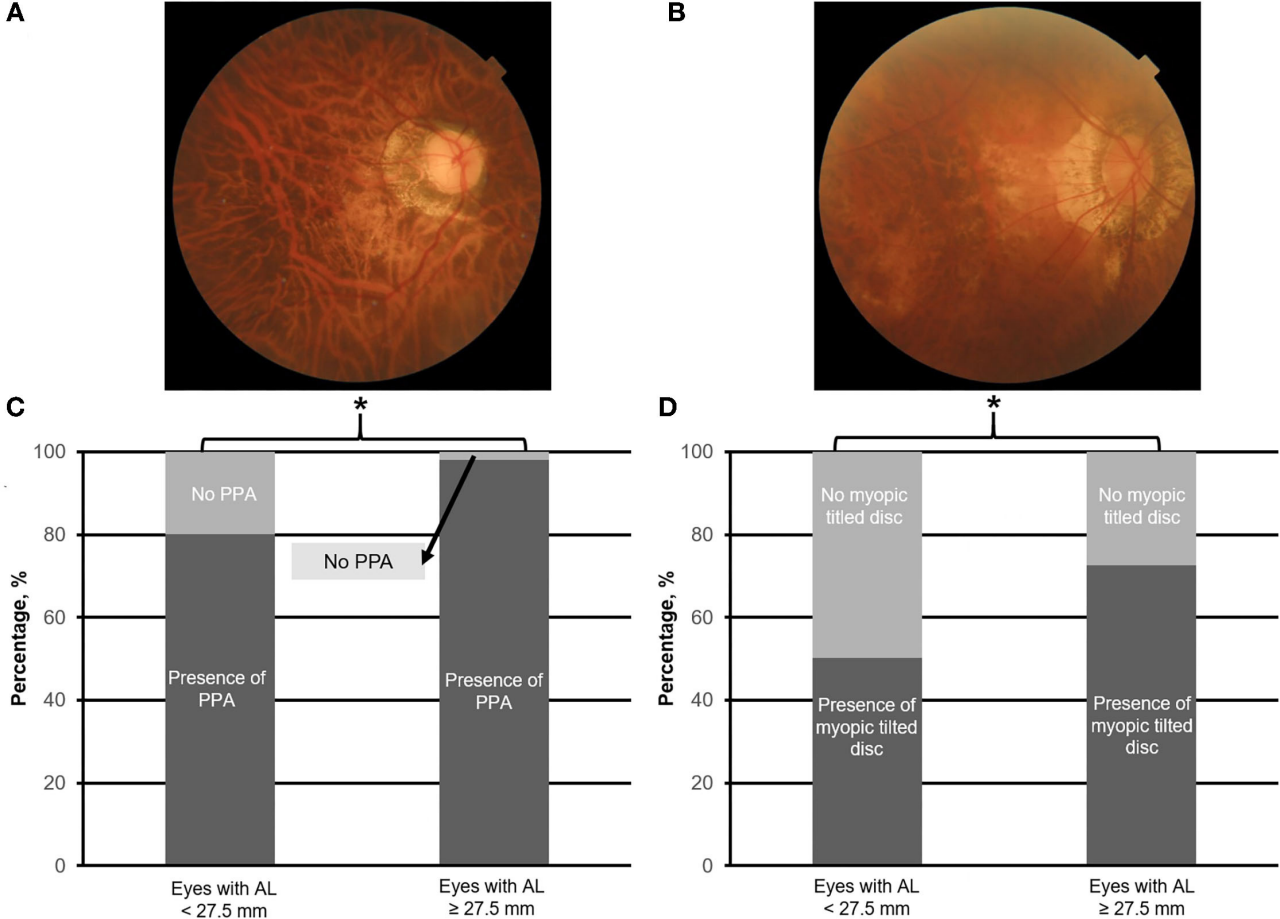
Top panels **(A,B)** are the representative fundus photographs of peripapillary atrophy (PPA; moderate) and myopic tilt disc (moderate), respectively. Bottom panels **(C,D)** illustrate the prevalence of PPA and myopic tilted disc in eyes with axial length (AL) 25.0–<27.5.0 mm vs. with AL ≥ 27.5 mm, respectively. Asterisk (*) denotes statistical significance (*p* < 0.05).

**Figure 3:**
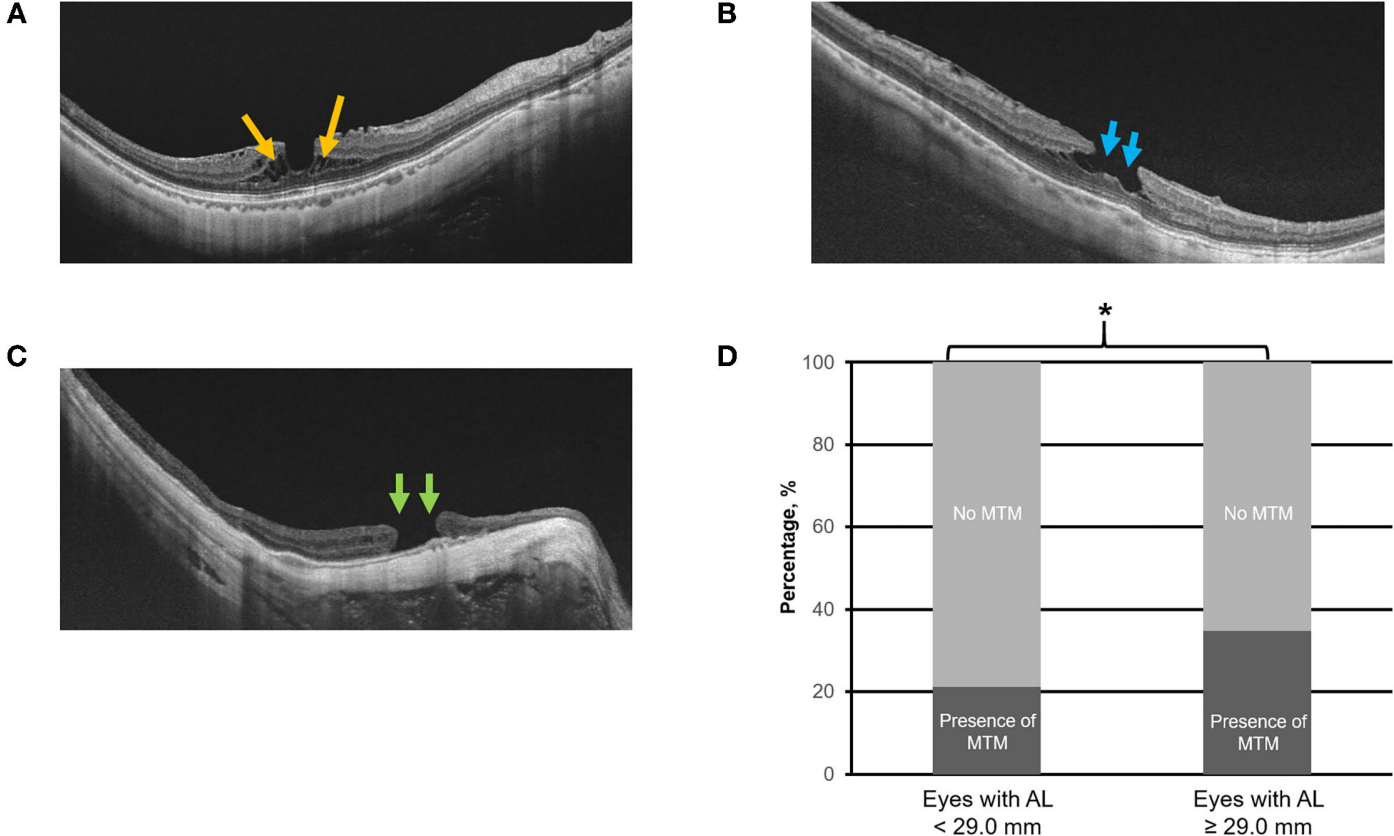
Panels **(A–C)** are the representative swept-source optical coherence scans of myopic traction maculopathy (MTM), e.g., myopic foveoschisis **(A)**, a partial-thickness macular hole **(B)**, and a full-thickness macular hole **(C)**. Panel **(D)** illustrates the prevalence of MTM in eyes with axial length (AL) 25.0–<29.0 mm vs. with AL ≥ 29.0 mm. Asterisk (*) denotes statistical significance (*p* < 0.05). Yellow arrows denote the area of foveoschisis. Blue arrows denote a partial-thickness macular hole. Green arrows denote full-thickness macular hole.

Specifically, fundus images were evaluated for the presence of the following: (1) MMD based on the international Meta Analyses of Pathologic Myopia (META-PM) classifications, where the presence of MMD was defined as META-PM MMD category 2 (diffuse atrophy) or worse (e.g., Figure 1A), or the presence of plus signs (e.g., lacquer cracks, mCNV, or Fuchs spots; Figure 1B) (6), (2) PPA (e.g., Figure 2A), and (3) myopic tilted disc (e.g., Figure 2B) defined as an optic disc with a ratio of minimal to maximal disc diameter of 0.75 or less and was assessed via the use of fundus photography (31). Given that Fuchs spot is considered a quiescent mCNV (32), and mCNV is one of the most commonly described features of PM (33), we included both active and quiescent forms in our prevalence of mCNV.

**Figure 4:**
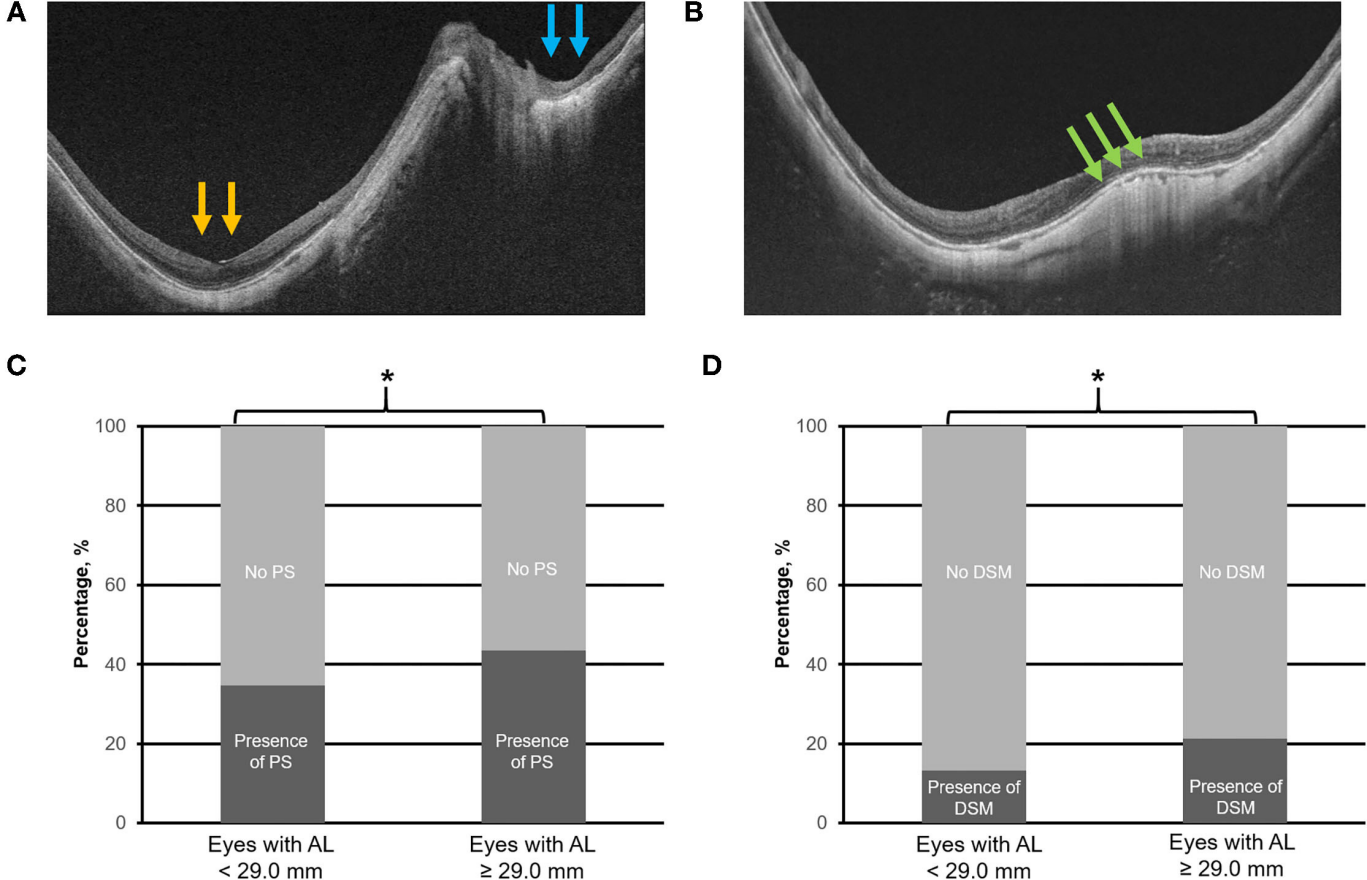
Top panels **(A,B)** are the representative swept-source optical coherence tomography scans of posterior staphyloma (PS; compound type—macular PS as denoted by the yellow arrows, and peripapillary PS as denoted by the blue arrows) and dome-shaped macula (DSM), respectively. Bottom panels **(C,D)** illustrate the prevalence of PS and DSM in eyes with axial length (AL) 25.0–<29.0 mm vs. with AL ≥ 29.0 mm. Asterisk (*) denotes statistical significance (*p* < 0.05). Yellow and blue arrows denote abrupt changes in the radius of curvature of the posterior sclera. Green arrows denote the inward bulge of the retinal pigment epithelium line.

**Figure 5:**
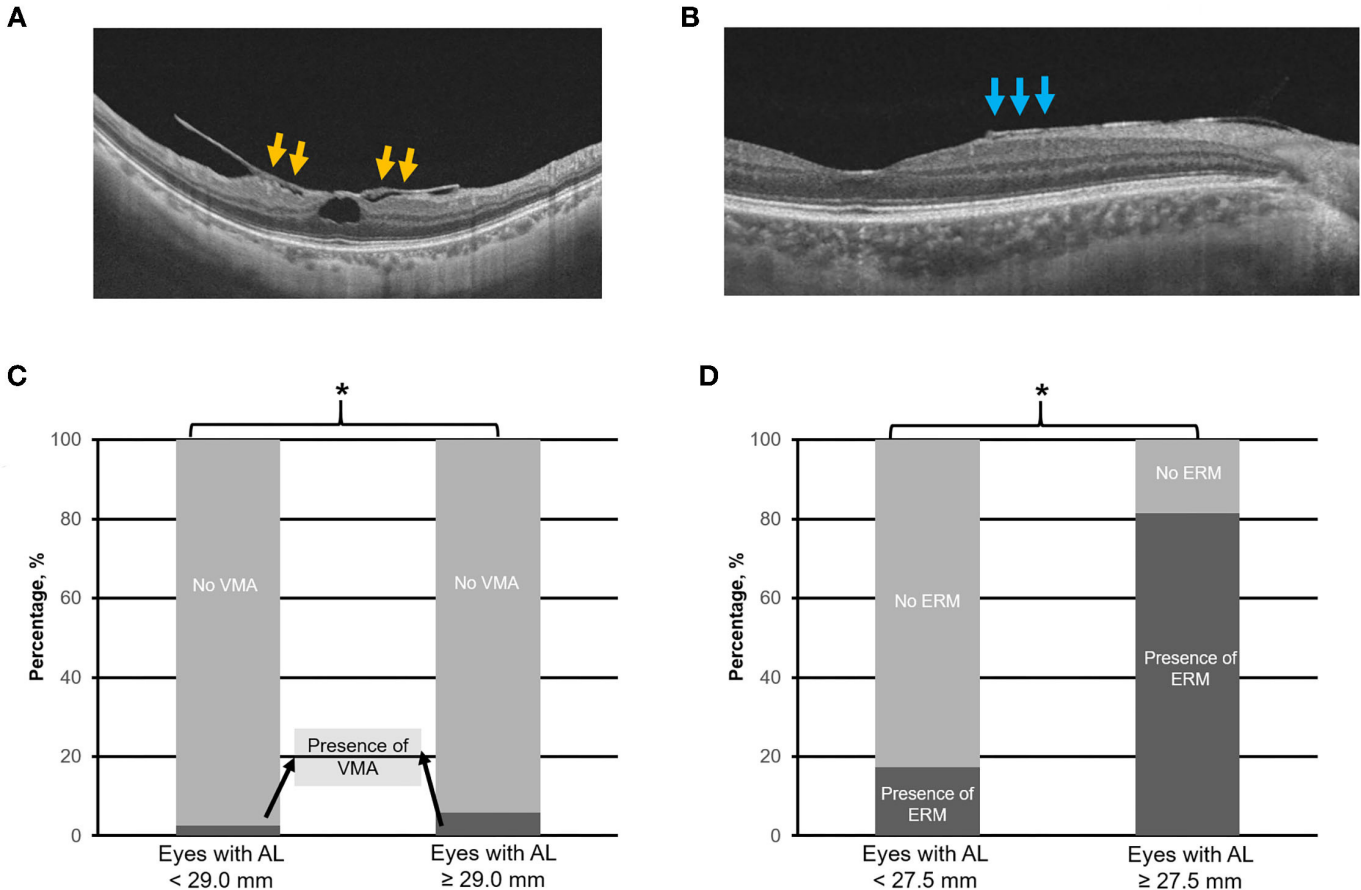
Top panels **(A,B)** are the representative swept-source optical coherence tomography scans of vitreomacular adhesion (VMA) and the epiretinal membrane (ERM), respectively. Bottom panels **(C,D)** illustrate the prevalence of VMA and ERM in eyes with axial length (AL) 25.0–< 29.0 mm vs. with AL ≥ 29.0 mm, and AL 25.0–<27.5 mm vs. with AL ≥ 27.5 mm, respectively. Asterisk (*) denotes statistical significance (*p* < 0.05). Yellow arrows denote VMA. Blue arrows denote ERM.

Swept-source optical coherence tomography scans were assessed for the presence of the following: (1) MTM (e.g., Figures 3A–C) defined by the presence of extrafoveal schisis, foveoschisis (with or without foveal detachment), or macular hole (partial/full-thickness macular hole, such as previously repaired macular hole) (10), (2) PS (e.g., Figure 4A) defined as an abrupt change in radius of curvature of the posterior sclera (34), (3) DSM (e.g., Figure 4B) defined as a hill-like appearance [an inward bludge of the retinal pigment epithelium (RPE)], greater than 50 μm on the most convex vertical or horizontal OCT sections above a presumed line tangent to the outer surface of the RPE, or at the bottom of the PS in HM eyes) (35), (4) VMA (e.g., Figure 5A), and (5) ERM (e.g., Figure 5B). In this analysis, we also included saddle-shaped macula (SSM, a hill-like surface present only in one meridian) (35) within the DSM group.

### Outcome Measures and Statistical Analysis

IBM SPSS Statistics for Windows version 26 (IBM Corp., Armonk, NY, USA) was used for the statistical analysis in this study. Our cohort of 923 eyes was arranged in order of AL and divided into four equal groups based on quartiles. Specifically, the quartile 1 (Q1) group contained eyes with AL ranging from 25.0 to 27.4 mm, quartile 2 (Q2: AL 27.5–28.9 mm), quartile 3 (Q3: AL 29.0–30.7 mm), and lastly, group 4 contained quartile 4 (Q4: AL 30.8–36.7 mm). The χ^2^-test was performed on adjacent quartiles (Q1 vs. Q2, Q2 vs. Q3, and Q3 vs. Q4) to screen for potential cut-off thresholds for significant differences in prevalence in pathologic findings as detailed above and instances where MMD and MTM (MMD+MTM), or MMD, MTM, and DSM (MMD+MTM+DSM) occurred concurrently.

Eyes were then stratified into two groups depending on the threshold identified above for various pathologies analyzed. For conditions in which two or more thresholds were identified, the lower threshold was employed. For conditions without a threshold identified, the Q2 cut-off (AL 29.0 mm) was employed. The mean differences in AL were analyzed with the independent *t*-test for between-groups comparison. Prevalence in each group of MMD, mCNV, MTM, PPA, myopic tilted disc, PS, ERM, DSM, VMA, and conditions occurring concurrently (such as MMD+MTM concurrently, and MMD+MTM+DSM concurrently) were compared using the χ^2^-test. These tests were two-sided with statistical significance set at *p* < 0.05.

## Results

Overall, 495 subjects were included in the study. A total of 990 eyes were imaged: 923 eyes were included in the analysis after excluding 45 unilateral eyes with AL <25.0 mm, and 22 eyes due to poor image signal or inability to obtain images. In total, 326 (65.9%) of included subjects were female, and the mean age was 61.7 ± 13.7 years old (range 19–91 years old). The overall mean AL was 29.2 ± 2.2 mm (range 25.0–36.7 mm).

Overall, there were 231 (25.0%) eyes in Q1, 231 (25.0%) eyes in Q2, 231 (25.0%) eyes in Q3, and 230 (25.0%) eyes in Q4. The mean ALs were 26.5 ± 0.6, 28.2 ± 0.5, 29.8 ± 0.5, and 32.3 ± 1.2 mm for Q1, Q2, Q3, and Q4, respectively. Characteristics of patients and a summary of the χ^2^-test analysis are found in Table 1. In summary, the identified threshold for MMD, PPA, myopic tilted disc, ERM, and for concurrent MMD+MTM was AL > 27.5 mm, whereas for MTM and for concurrent MMD+MTM+DSM, the threshold was AL > 29.0 mm. The summary of the χ^2^-test analysis based on the threshold identified is found in Table 2.

**Table 1:** Prevalence of pathologic changes in high myopia eyes stratified by axial length into quartiles.

			**Overall**							
**Patients**, ***n*** **(number of eyes included)**			495 (923)							
**Females**, ***n*** **(%)**			326 (65.9%)							
**Mean age, years old (range)**			61.7 ± 13.7 (19–93)							
**Axial length, mm (range)**			29.2 ± 2.2 (25.0–36.7)							
	**Q1**	**Q2**	**Q3**	**Q4**	**Q1 vs. Q2**		**Q2 vs. Q3**		**Q3 vs. Q4**	
***n***	**231**	**231**	**231**	**230**						
**AL range**	**25.0–27.4**	**27.5–28.9**	**29.0–30.7**	**30.8–36.7**						
**Mean AL, mm**	**26.5 ± 0.6**	**28.2 ± 0.5**	**29.8 ± 0.5**	**32.3 ± 1.2**						
	***n*** **(%)**	***n*** **(%)**	***n*** **(%)**	***n*** **(%)**	χ^2^	***p***	χ^2^	***p***	χ^2^	***p***
**MMD**	114 (49.4)	166 (71.9)	214 (92.6)	230 (100.0)	24.5	<0.001^*^	34.2	<0.001^*^	17.6	<0.001^*^
**mCNV or Fuchs spot**	22 (9.5)	34 (14.7)	39 (16.9)	41 (17.8)	2.9	0.87	0.4	0.524	0.1	0.789
**MTM**	41 (17.7)	58 (25.1)	88 (38.1)	72 (31.3)	3.7	0.054	9.0	0.003^*^	2.3	0.126
**PPA**	185 (80.1)	224 (97.0)	229 (99.1)	226 (98.3)	32.4	**<** **0.001** ^*^	2.8	0.092	0.7	0.408
**Myopic tilted disc**	116 (50.2)	157 (68.0)	162 (70.1)	184 (80.0)	15.1	**<** 0.001^*^	0.3	0.615	6.0	0.014^*^
**PS**	76 (32.9)	87 (37.7)	91 (39.4)	109 (47.4)	1.1	0.284	0.1	0.702	3.0	0.083
**DSM**	28 (12.1)	34 (14.7)	43 (18.6)	55 (23.9)	0.7	0.413	1.3	0.3	1.9	0.164
**VMA**	6 (2.6)	6 (2.6)	14 (6.1)	13 (5.6)	0	0.99	3.3	0.067	0.04	0.852
**ERM**	40 (17.3)	62 (26.8)	65 (28,1)	61 (26.5)	6.1	0.014^*^	0.1	0.755	0.2	0.697
**MMD**+**MTM**	27 (11.7)	51 (22.1)	84 (36.4)	72 (31.3)	8.9	0.003^*^	11.4	0.001^*^	1.3	0.251
**MMD**+**MTM**+**DSM**	5 (2.2)	8 (3.5)	25 (10.8)	18 (7.8)	0.7	0.399	9.4	0.002^*^	1.2	0.269

*AL, axial length; DSM, dome-shaped macula; ERM, epiretinal membrane; mCNV, myopic choroidal neovascularization; MMD, myopic macular degeneration; MTM, myopic traction maculopathy; PPA, peripapillary atrophy; PS, posterior staphyloma; Q, Quartile; VMA, vitreomacular adhesion*.

(*) *Asterisks and bold font represent statistical significance (p < 0.05)*.

**Table 2:** Summary of the χ^2^ analysis made between eyes with AL < 27.5 mm vs. AL ≥ 27.5 mm, and AL < 29.0 mm vs. AL ≥ 29.0 mm.

	**Eyes with AL <** **27.5 mm (*n* = 231)**	**Eyes with AL ≥** **27.5 mm (*n* = 692)**	**Pearson chi-squared** **value**	***p*** **-values**
**MMD**, ***n*** **(%)**	114 (49.4)	610 (88.2)	154.2	<0.001^*^
**PPA**, ***n*** **(%)**	185 (80.1)	679 (98.1%)	94.1	<0.001^*^
**Myopic tilted disc**, ***n*** **(%)**	116 (50.2)	503 (72.7)	39.6	<0.001^*^
**ERM**, ***n*** **(%)**	40 (17.3)	188 (81.4)	9.0	0.003^*^
**MMD**+**MTM**, ***n*** **(%)**	27 (11.7)	207 (30.0)	30.4	<0.001^*^
	**Eyes with AL** **<** **29.0 mm (*****n*** **=** **462)**	**Eyes with AL** **≥** **29.0 mm (*****n*** **=** **461)**	**Pearson chi-squared value**	***p*** **-values**
**mCNV (active mCNV and Fuchs spot)**, ***n*** **(%)**	57 (12.1)	80 (17.4)	5.0	0.03^*^
**MTM**, ***n*** **(%)**	99 (21.1)	160 (34.7)	55.8	<0.001^*^
**PS**, ***n*** **(%)**	163 (34.7)	200 (43.4)	6.3	0.012^*^
**DSM**, ***n*** **(%)**	62 (13.2)	98 (21.3)	9.9	0.002^*^
**VMA**, ***n*** **(%)**	12 (2.6)	27 (5.9)	6.1	0.014^*^
**MMD**+**MTM**+**DSM**, ***n*** **(%)**	13 (2.8)	43 (9.3)	17.2	<0.001^*^

*AL, axial length; DSM, dome-shaped macula; ERM, epiretinal membrane; mCNV, myopic choroidal neovascularization; mm, millimeters; MMD, myopic macular degeneration; MTM, myopic traction maculopathy; PPA, peripapillary atrophy; PS, posterior staphyloma; VMA, vitreomacular adhesion*.

(*) *Asterisks and bold font represent statistical significance (p < 0.05)*.

### Prevalence of MMD

Of 923 eyes, 724 (78.4%) eyes were found to have the presence of MMD. Overall, 9 (1.0%) eyes were found to have no MMD, 206 (22.3%) eyes were found to have tessellated fundus (Category 1 MMD), 401 (43.4%) eyes had diffuse atrophy (Category 2), 168 (18.2%) eyes had patchy atrophy (Category 3), and 139 (15.1%) eyes had macular atrophy (Category 4). For plus signs, 120 (13.0%) patients had Fuchs spot, 43 (4.7%) patients had lacquer crack, and 16 (1.7%) patients had active mCNV. The overall prevalence of MMD is found in Figure 1C.

The threshold identified for MMD was AL ≥ 27.5 mm (Q1 vs. Q2; χ^2^ = 24.5; *p* < 0.001, Table 1). Specifically, the prevalence of MMD was significantly higher in eyes with AL ≥ 27.5 mm, with 610 (88.2%) of the eyes found to have the presence of MMD, as compared to 114 (49.4%) in eyes <27.5 mm (χ^2^ = 154.2; *p* < 0.001; Table 2; Figure 1D). The prevalence of mCNV (active mCNV or Fuchs spot) was significantly higher in in eyes with AL ≥ 29.0 mm with 80 (17.4%) eyes found to have presence of mCNV, as compared to the 57 (12.1%) eyes of AL <29.0 mm (χ^2^ = 5.0; *p* = 0.03; Table 2; Figure 1D).

### Prevalence of MTM

Of the 923 images obtained, 259 (28.1%) eyes were found to have presence of MTM. The threshold identified for MTM was AL ≥ 29.0 mm (Q2 vs. Q3; χ^2^ = 9.0; *p* = 0.003, Table 1). Specifically, the prevalence of MTM was significantly higher in the in eyes with AL ≥ 29.0 mm with 160 (34.7%) eyes found to have MTM, as compared to the 99 (21.1%) eyes of AL <29.0 mm (χ^2^ = 55.8; *p* < 0.001; Table 2; Figure 3D).

### Prevalence of Other Associated Myopic Features

Overall, of the 923 images obtained, 864 (93.6%), 619 (67.1%), 363 (39.3%), 228 (24.7%), 160 (17.3%), and 39 (4.2%) eyes were found to have PPA, myopic tilted disc, PS, ERM, DSM, and VMA, respectively.

The identified threshold for PPA (Q1 vs. Q2; χ^2^ = 32.4; *p* < 0.001), myopic tilted disc (Q1 vs. Q2; χ^2^ = 15.1; *p* < 0.001), and ERM (Q1 vs. Q2; χ^2^ = 6.1; *p* < 0.003) was AL ≥ 27.5 mm (Table 1). The prevalence of PPA (χ^2^ = 94.1; *p* < 0.001; Figure 2C), myopic tilted disc (χ^2^ = 39.6; *p* < 0.001; Figure 2D), and ERM (χ^2^ = 9.0; *p* < 0.001; Figure 5D) was higher in eyes with AL ≥ 27.5 mm, as compared to eyes with AL <27.5 mm (Table 2). The prevalence of PS (χ^2^ = 6.3; *p* = 0.012; Figure 4C), DSM (χ^2^ = 9.9; *p* = 0.002; Figure 4D), and VMA (χ^2^ = 6.058; *p* = 0.014; Figure 5C) was significantly higher in the eyes of AL ≥ 29.0 mm when compared to eyes with AL <29.0 mm (Table 2).

**Figure 6:**
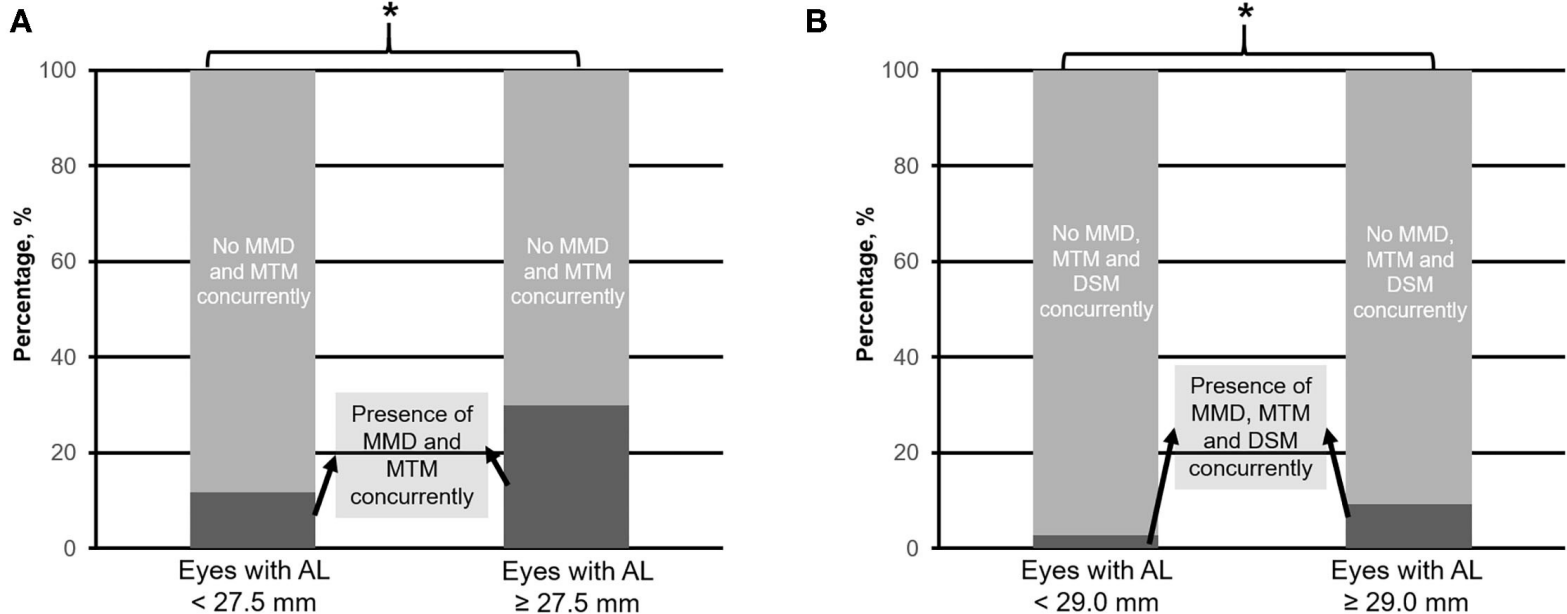
Prevalence of myopic macular degeneration (MMD) and myopic macular traction maculopathy (MTM) concurrently **(A)**, and MMD, MTM plus dome-shaped macula (DSM) concurrently **(B)** in eyes with axial length (AL) 25.0–<27.5 mm vs. with AL ≥ 27.5 mm, and 25.0–<29.0 mm vs. with AL ≥ 29.0 mm, respectively. Asterisk (*) denotes statistical significance (*p* < 0.05).

When considering eyes with multiple concurrent myopic pathologic changes, 234 (25.4%) eyes had MMD and MTM (MMD+MTM) concurrently (Figure 6A), whereas 56 (6.1%) eyes had MMD, MTM, and DSM (MMD+MTM+DSM) concurrently (Figure 6B). The identified threshold for MMD+MTM AL ≥ 27.5 mm (Q1 vs. Q2; χ^2^ = 8.9; *p* = 0.003, Table 1), while the threshold for MMD+MTM+DSM was AL ≥ 29.0 mm (Q2 vs. Q3; χ^2^ = 9.4; *p* = 0.002, Table 1). Prevalence of MMD and MTM concurrently was higher in eyes with AL ≥ 27.5 mm, with 207 (30.0%) eyes displaying MMD+MTM concurrently as compared to eyes with AL <27.5 mm, among which 27 (11.7%) eyes displayed MMD+MTM (χ^2^ = 30.4; *p* < 0.001, Table 2). Prevalence of MMD+MTM+DSM concurrently was significantly higher in eyes with AL ≥ 29.0 mm, with 43 (9.3%) found to have MMD+MTM+DSM concurrently, as compared to 13 (2.8%) eyes with AL <29.0 mm (χ^2^ = 17.2; *p* < 0.001, Table 2).

## Discussion

In this study, we established the prevalence of MMD, MTM, and associated myopic features in a cohort of hospital-based patients with HM using multimodal imaging. Overall, we observed that even among HM eyes, i.e., AL ≥ 25.0 mm, longer eyes were associated with a higher prevalence of MMD, mCNV, MTM, and associated myopic features, namely, myopic tilted disc, PPA, PS, DSM, ERM, and VMA when compared to relatively shorter HM eyes. Our quartile analysis suggested an AL threshold above which the prevalence of certain myopic pathologies was significantly higher, namely, for MMD (AL ≥ 27.5 mm), MTM (AL ≥ 29.0 mm), PPA (AL ≥ 27.5 mm), myopic tilted disc (AL ≥ 27.5 mm), MMD+MTM concurrently (AL ≥ 27.5 mm), and MMD+MTM+DSM concurrently (AL ≥ 29.0 mm).

The presence of MMD in HM eyes has been well-described in the literature. The prevalence of MMD has been reported to be 1.3–6.0% in the general population (36–45). Specifically, the prevalence of MMD among subjects with HM was observed to be 25.3–71.4%, which varies depending on the population reported (36–45). In our study, we observed a prevalence of 78.4% among hospital-based subjects with HM, which is slightly higher than the upper end of the previously reported number. This is likely attributed to the fact that this cohort of patients (MyoPES cohort) was recruited from a tertiary referral center (Singapore National Eye Centre High Myopia Clinic, Singapore), which specifically looks after high myopes, in which a vast majority have PM as compared to population studies that involve subjects from the general population. In addition, although eyes from both groups were considered to be HM, we observed that long HM eyes (AL ≥ 27.5 mm) were found to have a higher prevalence of MMD (88.2 vs. 49.4%; *p* < 0.001). This finding is consistent with Choudhury et al.'s findings, which found that longer AL is an independent risk factor for MMD (46). Similarly, this finding is also supported by the Hisayama study (47), which established an association between AL elongation and the likelihood of MMD. While AL has been identified as an independent risk factor for MMD development (48), the exact mechanism of what drives the pathology is unknown. Ohsugi et al. previously reported that AL was the greatest in those with mCNV (49), thus suggesting that the presence of MMD could be mainly driven by AL elongation. Our study shared a similar observation, where the prevalence of mCNV (both active and quiescent forms) was found to be significantly higher in eyes with longer AL (≥27.5 mm). We found a significantly higher prevalence of MMD and mCNV in HM eyes with AL ≥ 27.5 mm, and therefore, it is important to monitor such patients more frequently vs. eyes <27.5 mm.

Overall, we observed an overall prevalence of 28.1% for the presence of MTM among our HM eyes, which was similar to what was reported by Panozzo and Mercanti (34.4%) (9). Long AL has been a common theme in eyes with MTM (50, 51). In particular, we have found that the prevalence of MTM was significantly higher among eyes with extremely long AL, especially after the threshold AL of ≥ 29.0 mm. Similar observations were made by Xia et al. (50–54). Patients with MTM can have a prolonged subclinical phase in which they remain asymptomatic, and early changes can be difficult to diagnose on routine fundus examination (8). With OCT and an identified threshold, i.e., AL ≥ 29.0 mm, however, MTM can now be detected even in its early stages (55), enabling the timely diagnosis and treatment of patients at risk.

We also examined other pathological features that have been commonly described among HM eyes, as mentioned below. Peripapillary atrophy is more commonly seen in HM eyes (56, 57) and is an important feature to be identified given that it is not only a hallmark of HM but also an important factor for higher risk of glaucomatous damage (58). Myopic tilted disc and PS have also been reported to be progressive in nature in eyes with severe myopia (17, 59, 60). Axial length was identified as a risk factor for the presence of ERM (61), and similarly, DSM and VMA are conditions that are commonly seen in patients with HM (26, 27), and although these conditions alone might not affect visual function, they may increase the risk of developing further sight-threatening complications (26, 27). We found that the prevalence of PPA, myopic tilted disc, PS, DSM, ERM, and VMA was significantly higher in eyes with longer AL even among HM eyes. The association between the presence of these features and AL elongation has also been seen in other studies as well (26, 58, 62, 63). This suggests that AL elongation not only drives the pathogenic pathway of MMD and MTM but may in part lead to the development of these associated features as a by-product (24, 63–65). These patients should be followed up regularly with multimodal imaging for a comprehensive evaluation of these pathologies, and patients should be counseled about the symptoms of sight-threatening conditions, such as mCNV and MTM, to allow for early detection and appropriate interventions.

In this study, we also looked at the prevalence of accumulated eye conditions, namely, MMD+MTM concurrently and MMD+MTM+DSM concurrently. Overall, we found that MMD+MTM is of higher prevalence in eyes with AL ≥ 27.5 mm as compared to eyes with AL <29.0 mm. Our study findings concur with our previous study where we found that eyes with MTM are at higher risk of developing MMD (12), as the tractional changes may lead to stretching of retinal layers, which may be associated with degenerative changes seen in MMD. In this study, we have further established the threshold where the prevalence of both events occurs concurrently, which is an AL of 27.5 mm. Similarly, we see a higher prevalence of MMD, MTM, and DSM occurring concurrently in eyes with AL ≥ 29.0 mm as compared to eyes with AL <29.0 mm, and the implication of these findings would be interesting to be explored in a further longitudinal study.

The strengths of our study include the relatively large sample size with comprehensive multimodal imaging to accurately capture the prevalence of multiple HM-related pathologies. Our limitations include first, the cross-sectional design of our study precludes the analysis of incidence and causation. Ideally, a longitudinal study design would be preferred, although difficult in the context of a slowly progressive disease, such as MMD. Second, participants in our study are patients with HM recruited from a tertiary referral center, who are more likely to have worse pathology. Our results, therefore, may not be generalizable to the entire population of individuals with HM. Third, we are not able to establish the threshold of PS, DSM, and VMA, which is likely due to the relatively low prevalence of these phenotypes observed in our study group when subdivided into quartiles.Analyses of these parameters did reach significance when subdivided into two groups (Table 2).

In conclusion, our study described the overall prevalence of PM (MMD and MTM) and associated myopic pathological features in a cohort of hospital-based patients with HM. We found that longer eyes are more likely to have MMD, PPA, myopic tilted disc, ERM, and chances of MMD and MTM concurrently if AL ≥ 27.5 mm, and mCNV, MTM, myopic tilted disc, PS, DSM, VMA and chances of MMD, MTM, and DSM concurrently if AL ≥ 29.0 mm. We recommend a relatively closer follow-up with multimodal imaging for the timely diagnosis and treatment of sight-threatening complications in these extremely myopic individuals.

## Data Availability Statement

The raw data supporting the conclusions of this article will be made available by the authors, without undue reservation.

## Ethics Statement

The studies involving human participants were reviewed and approved by SingHealth Institutional Review Board. The patients/participants provided their written informed consent to participate in this study.

## Author Contributions

QH and CW conceptualized and supervised the study and obtained the data. KT, IL, YD, QW, DY, and VY curated the data and conducted formal analysis of data. All authors wrote, reviewed, edited, and approved the manuscript.

## Funding

This work was supported in part by the SingHealth-Duke-NUS Eye Academic Clinical Program Nurturing Clinician Scientist Scheme (NCSS/R1364/50/2016, CW, Singapore), the National Eye Institute/National Institutes of Health (Grant K08 EY023595, QH, USA), and the National Medical Research Council (Grant CSA/MOH-000151/2019, QH, Singapore). The funding organizations had no role in the design or conduct of this research.

## Conflict of Interest

The authors declare that the research was conducted in the absence of any commercial or financial relationships that could be construed as a potential conflict of interest.

## Publisher's Note

All claims expressed in this article are solely those of the authors and do not necessarily represent those of their affiliated organizations, or those of the publisher, the editors and the reviewers. Any product that may be evaluated in this article, or claim that may be made by its manufacturer, is not guaranteed or endorsed by the publisher.

## SNEC Retina Group

Anna Cheng Sim Tan, Beau James Fenner, Choi Mun Chan, Ian Yew San Yeo, Kelvin Yi Chong Teo, Ranjana Mathur, Shaun Sebastian Sim, Wiryasaputra Shaan, Andrew Shih Hsiang Tsai, Chong Lye Ang, Ning Cheung, Ting Shu Wei Daniel, Doric Wen Kuan Wong, Edmund Yick Mun Wong, Gavin Siew Wei Tan, and Hyungtaek Rim.
